# Perspectives on emotional memory images and the persistence of pain

**DOI:** 10.3389/fpain.2023.1217721

**Published:** 2023-07-26

**Authors:** Matt Hudson, Mark I. Johnson

**Affiliations:** ^1^ Centre for Pain Research, School of Health, Leeds Beckett University, Leeds, United Kingdom; ^2^ Mind Help Limited, Durham, United Kingdom

**Keywords:** persistent (chronic) pain, pain, Split-Second Unlearning, psychotherapeutic, psychophysiological dis-ease, intractable pain, emotional memory images

## Abstract

Multiple influences prevent recovery from pain. Our viewpoint is that non-conscious emotional memory images (EMIs) triggers outdated stress responses contributing to the intractability of pain. In this perspectives article we explore the concept that EMIs contribute to the persistence of pain. We contend that psychophysiological “stress” responses, resulting from first-time, novel and unprecedented pernicious or adverse events form EMIs within very short time frames (split-second learning). Subsequently, these EMIs are re-triggered in daily living, “re-playing” stress responses. We postulate that EMIs continually “raise the alarm” to socio-ecological stimuli by re-triggering the HPA-axis and amplifying neural input associated with threat, fear, anxiety, and pain, creating a debilitating state of psychophysiological dis-ease. We position the EMI within a philosophical debate on the nature and locus of memory and explain how the EMI, irrespective of whether it is a “thing” or a metaphor, can create a basis of understanding for the client to grasp. We describe a therapeutic approach (Split-Second Unlearning) to “clear” EMIs and the “stickiness” of pain and help people embark on a healing journey. This involves surveillance of clients for micro-expression(s) signifying an in-the-moment stress response, representative of the presence of an EMI, and encouraging the client to become a curious observer within/of their own experience. This helps the client detach their EMI from its stress response. We contend that this occurs rapidly without the need to get bogged down in a whole-life narrative. We advocate further exploration of our EMI model of dis-ease in the context of intractable pain.

## Introduction

Intractable pain that persists beyond the expected time for recovery affects a large proportion of people and is burdensome on society (1). In 2018, Borsook et al. (2) introduced the concept of “pain stickiness” as a nickname to capture multiple influences preventing recovery from pain, i.e., being stuck in pain despite therapeutic intervention. Borsook et al. explored reasons why some people engage adaptive responses to a perturbation (e.g., physical trauma, surgery or disease) enabling recovery, whereas others do not. Borstook et al. argued that neurobiologically informed psychotherapy, focusing on pain as a motivational drive to avoid harm, would assist people to overcome maladaptive fixed pain behaviour.

Our viewpoint is that exploring pain stickiness through a psychoeducational lens offers opportunities to better understand the intractable nature of pain, and possible strategies to aid recovery. Previously, we proposed a psychoeducational model of “dis-ease” based on evidence that traumatic emotional events earlier in life block a person's ability to overcome maladaptive thoughts and behaviours later in life. This results in a state of psychophysiological stress (dis-ease) and a variety of symptoms, including intractable pain (3). We proposed that psychophysiological “stress” responses resulted from first-time, novel and unprecedented pernicious or adverse events. This formed emotional memory images (EMIs) within very short time frames (Split-Second Learning), and these EMIs are re-triggered in daily living “re-playing” stress responses (3, 4).

Our theory positions EMIs as a barrier to a person “moving forward”. We offered a technique to “unlearn” the EMI and aid recovery, i.e., Split-Second Unlearning. This involves screening clients for the presence of EMIs and placing the client as a curious observer within their own experience. In doing so, the client is able to “detach” (uncouple) the EMI from their stress response (dis-ease) so they can become naturally adaptive again.

In this perspectives article, we explore long-term intractable pain through an EMI lens. Our viewpoint is that emotionally overwhelming experiences, real or imagined, induce, non-conscious, contiguously formed multimodal mental imagery. This can trigger amnesic, anachronistic, stress responses within a split-second that may contribute to the intractability (stickiness) of pain. Our intention is to describe how Split-Second Learning informs a broader understanding of intractable pain and how our model of Split-Second Unlearning offers opportunities for therapeutic approaches.

## Context

Our Split-Second Unlearning model of psychophysiological dis-ease offers a new perspective on nebulous conditions, such as stress, anxiety, and pain that persists (e.g., chronic primary pain) (3). In brief, we proposed that physiological stress responses (i.e., sympathetically mediated) from first-time, novel and unprecedented “traumatic” emotional experience are learnt within a “split-second” and can be re-triggered later in daily life when a person encounters a “reminder”. These “reminders” may be pernicious or benign events that re-trigger latent non-conscious EMIs. EMIs activate a sympathetically-mediated stress response, producing bodily sensations associated with fight-flight-fright-freeze-flop (e.g., rapid heart rate, shallow breath, and sweaty palms). The sympathetic response is like an “echo” of the original archaic trauma (adverse event).

In the modern world people often appraise such sensations as negative emotional states detrimental to health and wellbeing, e.g., pain of sinister origin producing anxiety and fear. The cumulative effect of re-triggering EMIs is low-level psychophysiological stress, hypothalamic-pituitary-adrenal (HPA) axis dysregulation and dis-ease. Our appraisal of the attributes of EMIs suggested that the concept of EMI was distinct from other entities described in psychology literature e.g., emotional memory, mental image(ry), mental representation etc. [see Hudson and Johnson (4) for review].

We proposed that people “access” EMIs during conversation and that this manifests as non-verbal, non-conscious, momentary micro-expressions, e.g., sharp peripheral peek movements of the eyes that focus on the same exact spot whilst the client chats about their presenting problem. We suggested micro-expressions that signify a non-conscious “freeze-like” response may be used as non-verbal cues to prompt the client to curiously observe and explore their in-the-moment experience. By recognising the EMI as a barrier to moving forward, the client can engage with observable fragments of their response to “triggers”; this helps to detach the EMI from psychophysiological stress so that they can become naturally adaptive again, i.e., Split-Second Unlearning. Uncoupling traumatic memory and the associated stress response reduces a person's allostatic load with positive consequences for health and well-being (5).

Our Split-Second Unlearning theory of psychophysiological dis-ease (distress) is relatively simplistic, and we emphasise that our model in no way reduces the persistence of pain to one causal mechanism. Nevertheless, psychological distress (depressive and anxiety-related symptoms), is a risk factor for the persistence of pain and is correlated with increased pain prevalence (6–8). In the next section, we explore how EMIs could influence the persistence of pain.

## Learning and pain persistence: the role of EMIs

People learn the concept and construct of pain through life experience; thus pain is strongly influenced by social circumstances, i.e., past, present, and possible future events (9–11). Western medicine's deductive philosophical processes have to some extent, fostered a division between body and mind as separate entities, encouraging a biomedical model of pain that focuses on tissue at the expense of lived experience.

In biomedicine pain is defined as a subjective experience anchored to tissue, e.g., “*An unpleasant sensory and emotional experience associated with, or resembling that with, actual or potential tissue damage*” (12). Phenomenological definitions of pain tend to emphasise a fusion of body and mind and something that is familiar between people, e.g., “*Pain is a mutually recognisable somatic experience that reflects a person's apprehension of threat to their bodily or existential integrity.*” (13) p.6. Debates about the nature of pain as an entity (“thing”) (14), a type of event (15), or something else (16), including associations with bodily and extracorporeal processes are long-standing and unresolved (17).

People learn how to conceptualise and experience pain from childhood. This involves coupling bodily sensations and emotions existing in time and space to the word “pain”, under the influence of societal behaviours, narratives and norms (9, 11, 18). Pain is related to the ontological experience of being in a body, i.e., the embodied mind, conceptualised and narrated in language, influenced by environments, intersected by time, place and culture (19–22). Thus, pain, and its persistence, is a personal construct under the influence of a multidimensional array of interacting biopsychosocial factors. Contemporary models of pain management advocate a biopsychosocial approach grounded in contemporary pain science education, based on the principles of sensitisation and bioplasticity, to reconceptualise a person's view about persistent pain (23). Concepts at the core of pain science education include pain acts to protect, the “pain system [sic]” can become overprotective (hypersensitive), and a hypersensitive “pain system [sic]” can be “retrained” to work “normally” (24, 25). Lumley et al. (26) argue that trauma is treated to facilitate remission or recovery, whereas persistent pain is managed so that a person is better able to function with pain. Thus, Lumley et al. advocate pain science education to promote understanding of the role of brain processing (bioplasticity) in linking trauma and persistent pain, thus aiding recovery; meta-analyses evaluating the efficacy of pain science education are inconclusive due to a paucity of large robust clinical trials (27, 28).

Perceived threat, often associated with specific emotional episodic memories, is a key feature of persistent pain and anxiety disorders (29). Post-traumatic stress disorder, adversity, and emotional regulation is associated with central nervous system processes and brain function abnormalities, e.g., in the cingulate gyrus, inferior parietal lobule, and precuneus (30). Evidence suggests that in later life, detrimental early life conditions and adverse childhood events (ACEs) are associated with increased pain severity, persistence, and complications (31–37). Functional somatic syndromes, including chronic primary pain with central sensitisation (fibromyalgia, chronic widespread pain, irritable bowel syndrome etc.), is associated with post-traumatic stress disorder-type events (38, 39). In addition, pain is associated with adversity from more common distressing experiences that occur throughout life owing to social conditions including neglect, family discord, abuse, social injustice and national displacement (40).

Our viewpoint is that psychological trauma (adversity) may occur at any juncture in life, resulting in the formation of an EMI; the key is that the trauma is first-time, novel and unprecedented. For example, we speculate that EMIs produced in adults due to trauma associated with uncertainties and distress experienced by global populations during Covid-19, [e.g., lockdown, job insecurity, social isolation etc (41, 42).] contribute to the rise in Covid-19-related distress (dis-ease) (43), including new onset and persistent (stickiness) of pain (44).

Psychological therapy-based treatment is recommended for people living with intractable pain, yet high-certainty evidence of clinically meaningful benefit remains elusive. The most recent Cochrane review of 59 studies (>5,000 participants) provided moderate evidence of small or very small beneficial effects of Cognitive Behavioural Therapy (CBT) for reducing pain and distress in persistent pain (45). Evidence of benefit for Behaviour Therapy (BT, 8 studies, 647 participants) or Acceptance and Commitment Therapy (ACT, 5 studies, 443 participants) was less certain and judged to be of moderate to very low quality. In 2017, Eccleston and Crombez (46) contended that the development of psychological treatment had stalled and they advocated a “… radical re-imagining of the content, delivery, place, and control of therapy” p.1.

For some people with pain there is a “psychological barrier” to moving forward, even following pain science education and conventional psychological interventions. Our viewpoint is that EMIs have a critical role in this stickiness of pain. We defined an EMI as “*Trauma induced, non-conscious, contiguously formed multimodal mental imagery, which triggers an amnesic, anachronistic, stress response within a split-second.*” (4) p.1. We posit, EMIs are created when a person experiences a situation perceived to threaten bodily or existential integrity. For example, a person who has experienced a dog bite may develop fear and anxiety of all dogs. The EMI generalises a threat to bodily or existential integrity across time and space (place). Fear and anxiety may be learnt through observation of others. For example, an infant witnessing a parent in fear when encountering a spider may develop an EMI themselves, causing fear and anxiety of spiders. A vast array of pernicious or adverse experiences in early life, including learned behaviours and social modelling, may generate EMIs that trigger contextual fear and anxiety. Thus EMIs, when re-triggered later in life, catalyse a stress response mediated by HPA-axis activation and the release of hormones and neuromodulators (e.g., cortisol and adrenaline). This causes symptomology associated with fear, anxiety, palpitations, muscle tension, shortness of breath, and sensitivity to stimuli that mediate pain.

We argue that people are unaware of EMIs (i.e., non-conscious), leaving them oblivious to precipitating stimuli that cause a sense of “threat”, despite no apparent danger being present. Re-triggering of EMIs through exposure to stressors of modern life and chronic activation of the HPA-axis creates allostatic overload and a debilitating state of dis-ease comprising psychophysiological stress, anxiety, apprehension, and fear (47). Over time people may generalise anxiety and fear to other situations expanding precipitating circumstances beyond the scope of the original trigger i.e., stimulus generalisation (48).

The resultant allostatic overwhelm leads to dis-ease and intensification of pre-existing modern-day afflictions, (e.g., non-communicable disease) promoting behaviours to avoid situations that trigger further fear, anxiety, distress, discomfort, and pain (47, 49). Avoidance behaviour promotes a cycle of reinforcement, where the individual avoids situations that exacerbate anxiety and fear, increasing the likelihood of avoidance and further distress (dis-ease) (50, 51). As the dominant societal narrative is biomedical in nature people, appraise symptomology as medical (pathological) in origin and seek support from health care services that provide biomedical and/or psychological interventions.

Our viewpoint is that EMIs are grounded in a person's social context, past, present, and possible future. Consequently, EMIs serve to amplify the detrimental effects of social and economic risk factors for health, including persistent pain, such as family disruption, poverty, violence, crime, social isolation, and diminished economic opportunities. By exacerbating the impact of social risk factors on health and well-being, EMIs promulgate dis-ease, disability, and suffering, and hinder a person's “healing journey”.

The World Health Organisation (WHO) advocate a salutogenic approach to health and well-being by acknowledging the need to address socio-ecological risk factors for health and well-being, through, for example, a whole system healthy settings approach. Policymakers acknowledge the need for integrative health care services and interdisciplinary teams that adopt a biopsychosocial approach to manage persistent pain. This includes helping clients to understand the psychological effects of pain and improve confidence to cope with pain, as well as the importance of movement, pacing, relaxation of body and mind, and strategies to manage everyday activities, such as hobbies and work. At the practitioner level, we advocate consideration of the Split-Second Unlearning model as a framework to “clear” EMIs and help people with persistent pain “move on” (heal).

## Split-Second Unlearning and pain persistence

We postulate EMIs are formed (learned) in a split-second and hinder adaptation to the stressors of daily living, forming a barrier to recovery from pain. Our Split-Second Unlearning model (3) describes a novel psychotherapeutic approach to clear a client's EMI. This involves surveillance of clients for non-conscious “freeze-like” micro-expression(s) that signify an in-the-moment stress response, representative of the presence of an EMI. Encouraging the client to become a curious observer within/of their own experience, feeding back the non-verbal cues as they arrive in the moment, assists interruption of the informational flow of observable fragments, helping to detach their EMI from their psychophysiological stress response. We contend that this occurs rapidly without the need to get bogged down in a whole-life narrative.

Our psychotherapeutic approach has evolved from Eye Movement Desensitisation and Reprocessing (EMDR) (52). Gaze behaviour, where a person intensifies or averts gaze, is a behavioural strategy to regulate emotions and cope with stressful situations (53). We hypothesise that gaze behaviour may be associated with avoidance of, or fixating on, EMIs within the “mind”s eye”. EMDR is used to treat various conditions, including trauma and persistent pain, with evidence of physiological changes to support observations of clinical benefit (54, 55). Nevertheless, systematic reviews and meta-analyses evaluating the benefits and safety of EMDR interventions for persistent pain and post-traumatic stress disorder are inconclusive due to insufficient high-quality studies (56–60).

Our approach is based on EMDR and posing open questions such as “What would you like to work with today?” or “What is troubling you at the moment?”. These questions provoke the client to scan, in a non-conscious manner, memories in relation to their problem (e.g., pain) prior to formulating a conscious verbal reply. The therapist observes non-verbal micro-expressions, e.g., a sharp intake of breath, head tilt, pupil dilation and/or eyes making a sharp peripheral peek or fixating on a specific point in space. These occur in a split-second and indicate an emotional connection between a thought and a reflex stress response as if the client is re-experiencing some event from the past. These micro-expressions, of which eye movement and fixation are of importance, are indicative of a troubling EMI.

It is the connection between the EMI and the associated stress response that the therapist seeks to break. This is achieved by making the client curiously aware of their involuntary micro-expressions, such as fixation of eyes in a specific peripheral peek that appear each time they are asked about their presenting problem. States of curiosity enhance the capacity to learn new information such as dispassionate acceptance, this can break associations between emotions and reflex stress responses which no longer serve a useful purpose (61). The therapist uses various techniques to help the client uncouple the EMI from the stress reflex (i.e., unlearning), such as asking the client to direct their gaze to a different position while still trying to think about their problem.

Split-Second Unlearning refers to a brief window of opportunity in which the therapist observes the activation of the EMI. They then deploy an interruption to disconnect the non-conscious memory from the reflex stress response, replacing it with a more objective appraisal of the overall situation. Thus, the EMI may be deemed unimportant or infused with a clarity of hindsight. The “uncoupling” of an EMI to a stress response is usually “immediate” and recognised as (emotional and cognitive) confusion. Longer-term benefit arises from a stress response that is no longer re-triggered by the EMI, enabling the person to embark on a journey to recovery. This approach differs from conventional psychotherapeutic interventions because it does not encourage clients to share their personal narratives, simply to explore their experience within the moment.

### Clinical vignettes

The Split-Second Unlearning model offers a framework for practitioners to diagnose and treat EMIs born out of adversity. MH has used it successfully in a variety of conditions presenting with persistent pain including dysmenorrhea, irritable bowel syndrome, fibromyalgia, migraine, rheumatoid arthritis, and neuropathic pain. Here, MH describes two cases as examples of Split-Second Unlearning in practice.

#### Case 1 - dysmenorrhea

A 34-year-old female presented in an online clinical session reporting long-standing severe period pain that started at menarche. When the client described her pain story, I noticed that her eyes moved to the left when speaking of past events and to the right when speaking of future events. Rather puzzling to me was the observation that the client's eyes remained fixated to the right when describing period pain, irrespective of describing the past or future. I explained that first-time emotionally overwhelming events can lead to the creation of EMIs that remain within the mind and invisible to the person. I explained that EMIs can trigger similar “stress” responses to encounters with similar contexts and that feelings of vulnerability, guilt, shame, embarrassment, and being dirty or unclean, can rapidly create emotional overwhelm. The client nodded her head in agreement. I described Split-Second Unlearning and directed the client to fixate her eyes on my hand (central field of vision) and think about her period pains while I moved my hand back and forth for a few seconds. Whilst doing this the client looked a little confused and commented that “Something was different”, and I noticed her eyes were moving freely without any eye fixation or avoidance; I surmised the EMI had been effectively erased. The client was given an appointment for an online follow-up call at 1 month at which she reported no recurrence of symptoms. There were no symptoms at the 12-month follow-up.

#### Case 2 – trigeminal neuralgia

A 43-year-old male presented in an online clinical session with trigeminal neuralgia. The client reported first onset of pain at age 16 that was intractable and resistant to various treatments. This included a rhizotomy at age 25 and prescription medication that included carbamazepine, gabapentin, baclofen, and ibuprofen. These interventions provided only partial short-term relief. The client had been able to hold down a full-time job, marry and raise children. As the client told his story, I noticed that he continually fixated his eyes on a spot in his left peripheral field of vision. The client accessed the same spot when I asked what was happening in his life just before 16, and he replied, “My mum and dad got divorced”. For a fleeting moment the client's face flushed red. I described EMIs and Split-Second Unlearning and pointed out that he was continually accessing an EMI “on his left”. I invited the client to fixate his gaze on me (centre) and to re-tell the history of his pain (i.e., pain story); almost immediately the client smiled and said, “It's gone!”. I surmised that the process had, in a split-second, interrupted triggering of the EMI and in doing so broke the connection between the EMI (stimulus) and the stress response. I asked to be kept informed of any changes. The client reported being pain-free at follow-up contacts of 1, 3, 6 and 12 months.

### A rationale for the speed of Split-Second Unlearning

The process of Split-Second Unlearning aligns with principles of memory reconsolidation by:

1. Reactivating the client's awareness of the EMI.
2. Pointing to the EMI as the source of their pain, giving fresh insight into the experience.
3. Embellishing stages 1 and 2 to stimulate the process of unlearning, nullifying, and reconfiguring the EMI.

For further insight see (62, 63).

## Discussion

People visit health care professionals expecting to receive a physical diagnosis and biomedical (physical) treatment to “fix” all types of pain, including pain that has become intractable. This poses a challenge for practitioners trying to explore with their clients, psychosocial or metaphysical factors that may be influencing the persistence (stickiness) of pain. Biomedical interventions that “fix” pathology and/or facilitate symptomatic relief has revolutionised the management of painful conditions and the quality of life. Nevertheless, there remains a treatment-prevalence paradox whereby increasing varieties of biomedical and psychological interventions have not reduced the prevalence of persistent pain.

Foell states: “*It would be so easy and straightforward if persistent pain could be a thing. Pain without a lesion is a condition charged with moral judgement. … But, unfortunately, pain is not a thing. ‘Pain does not emerge naturally from physiological processes, but in negotiations with social worlds’* (15)” (64) p.126–127. Agarwal's “ecology of wholeness” model of chronic illness and the body in pain conceptualises pain according to the self (reflexive and embodied), the body (material and conversational) and the context (including body/self-integration, food, nature, time, change, illness intrusion and information). Contemporary neurophysiology suggests that pain emerges from predictive processing in the brain informed by multisensory input that “threatens” the integrity of the body and peri-personal space, at tissue and psychological levels (65). Moseley et al. (65) name coarse neural representation of the body and peri-personal space as the “body-matrix”, and suggest that disruption of the integrity of the body matrix by damage, malfunctioning or anomalous feedback, may drive various functional and psychological disorders including persistent pain. It is not our intention to debate the reification of pain, but rather to draw attention to the consequence of people being socialised to believe that pain is a “thing” that always results from tissue damage.

Pain and EMIs are positioned within a philosophical debate on the nature and locus of memory, and whether memory is an entity, phenomenon, or something else. The dominant neurophysiological, synaptic-plasticity theory of memory has been critiqued [e.g. (66)] and defended [e.g. (67)]. Deconstruction of the body within the reductionist framework of the Standard Model of Physics at organ, tissue, cellular, molecular, and subatomic levels has failed to resolve this debate. Thus, we do not constrain EMIs to be solely dependent on brain function and neural connections but indigenous to “the self”, and possibly extracorporeal (3, 4).

Practitioners may be afraid to step into the vulnerable space of discussing trauma and the metaphysical aspects of the EMI, as they fear reprisal from the client who expects a pathological cause and a biomedical treatment. Engaging the client in a discussion around the EMI, irrespective of whether the EMI is considered a “thing” or a metaphor for how the client's pain exists can create a basis for understanding for the client to grasp. For example, engaging the client's curiosity by discussing the possibility of the EMI acting as a metaphysical cloud storage at the intersection between the body and the socio-ecological context (external environment).

As a concept, this could have significant implications for understanding the intractability of many psychophysiological dis-eases, including persistent pain. Moreover, it could inspire new therapeutic approaches that incorporate both mind-based and body-based techniques. For example, the Split-Second Unlearning process has been integrated with eye-tracking technology to create “MindReset” a digital intervention that can be accessed through a mobile phone, with the potential for rapid, cost-effective and scalable “treatment”.

## Conclusion and next steps

In summary, we postulate that EMIs may contribute to the stickiness of pain, continually “raising the alarm” by re-triggering the HPA-axis in response to socio-ecological stimuli, i.e., a sensitised threat/fear system that in turn amplifies pain and suffering and blocks “recovery”. We postulate that the EMI is non-conscious, shrouding the original emotional overwhelm (trauma and adversity) in amnesia, so people are unable to verbalise the origin of their persistent and intractable pain, only that they have it and “cannot get rid of it”.

In conclusion, we advocate exploration of the persistence (stickiness) of pain through the lens of EMIs, psychophysiological dis-ease, and Past Adversity Influencing “Now” (PAIN). We plan to integrate EMIs with psychological (68), social communitive (10), and ecological (69) models of persistent pain. We suggest clinical research focuses on the utility and efficacy of the Split-Second Unlearning technique to (i) reveal pre-verbal trauma in people living with persistent pain; and (ii) alleviate the persistence of pain and related symptoms.

## Data Availability

The original contributions presented in the study are included in the article/Supplementary Material, further inquiries can be directed to the corresponding author.
